# Bureau of Materia Medica and Provings, I. H. A.

**Published:** 1889-02

**Authors:** E. A. Ballard

**Affiliations:** Chairman, 97 Thirty-seventh Street, Chicago, Ill.


### BUREAU OF MATERIA MEDICA AND PROVINGS, I. H. A.

P. P. Wells, M. D., Thos. Skinner, M. D., Edward Mahoney, M. D., E. W. Berridge, M. D., J. V. Allen, M. D., C. C. Smith, M. D., H. C. Allen, M. D., E. B. Nash, M. D., H. P. Holmes, M. D., Alice B. Campbell, M. D., Flora A. Waddell, M. D.

It is to be hoped no one will feel slighted because he has not received a written invitation to become a member of this bureau. Every member not pledged to another bureau is hereby invited to send a paper for next June, unless he intends to be present. The bounden duty of every member is to be at the meeting loaded with a paper of practical value. If it is impossible for him to be there, he should see that he is represented by his paper.

E. A. BALLARD, M. D., *Chairman*,
97 Thirty-seventh Street, Chicago, Ill.

